# P59 Growing up in rheumatology: An Evaluation of a Young Adult Rheumatology Service

**DOI:** 10.1093/rap/rkac067.059

**Published:** 2022-09-28

**Authors:** Emily Tiffney, Emily Willis, Ellen Bruce, Janet McDonagh

**Affiliations:** University of Manchester Medical School, Manchester, United Kingdom; Royal Manchester Children's Hospital, Manchester, United Kingdom; Manchester University Hospitals NHS Trust, Manchester, United Kingdom; Centre for Epidemiology Versus Arthritis, University of Manchester, Manchester, United Kingdom; NIHR Biomedical Research Centre, Manchester University Hospitals NHS Trust, Manchester, United Kingdom

## Abstract

**Introduction/Background:**

Young people with juvenile onset rheumatic disease are treated in paediatric centres until they are required to transfer to an adult centre. Transitional care is a process which begins in early adolescence and prepares young people for this change. There is a rapidly evolving evidence base to support transitional care for young people with long term health conditions with European rheumatology-specific guidance available. The third stage of transitional care, i.e. that which follows transfer to adult care, however, is the least researched.

**Description/Method:**

The aim of the study is to evaluate rheumatology service provision for the third stage of transition, i.e. young adult rheumatology provision, at a large NHS trust. A retrospective case-note review was conducted. Relevant patients were identified from young adult rheumatology clinic lists from 2017 to 2022. Of note, no clinics took place during the COVID 19 pandemic lock down in 2020 and transfer of patients during that time was postponed until face to face clinics resumed in adult practice. Patients were assigned study numbers and data was then collected from clinical letters, general correspondence and other annotations uploaded to the Electronic Patient Records (EPR). Patient records searched included those up to 24 years old on current adult rheumatology clinic lists.

**Discussion/Results:**

86 patients (79.1% female, n = 68) were identified. The mean age at transfer was 17.1 years with 53.4% having inflammatory arthritis (n = 46). 59.3% (n = 51) had more than 1 diagnosis. 64% (n = 55) had no other named specialties involved in care at transfer. The median number of medications at transfer was 3, range 0-11. Of those taking medications, 86.7% (n = 72) were self-managing these.

59.3% (n = 51) had a transfer letter. 29.1% (n = 25) had a transfer summary completed. 14% (n = 12) had no transfer documentation. Transition readiness checklists were identified in 41.9% records (n = 36). At the time of transfer, copy clinic letters were addressed solely to parents in 4 (4.7%).

Young people had attended a median of 5 appointments (range 1-12) in the 12 months prior to transfer. 59.3% (n = 51) attended more than 50% of these appointments independently. 26.7% (n = 23) did not attend between 1-2 of their scheduled appointments in the 12 months pre-transfer.

The mean duration between last paediatric and first adult appointment was 4 months (range 1-12). 47.7% (n = 41) did not attend at least one appointment in the 12 months post-transfer, with 23.3% (n = 20) not attending 3 or more appointments.

67% (n = 58) of the final paediatric letters covered at least one of 11 psychosocial topics, with a median of 2 topics covered (range 1-5). In the first adult clinic letter a median of 3 topics (range 1-7) were covered in 82.6% (n = 71) letters.

Around the time of transfer, disease activity was addressed in only 18 patients (20.9%) with a median score for Physician Global Assessment of 0 (0-10). 36% of patients (n = 31) had a CHAQ score documented, median score 0.5 (0-1.875). 29.1% (n = 25) had pain scores recorded, median 16, range 0-87. Self-reported wellbeing scores were recorded for 23.3% (n = 20), median 16.5, range 0-75.

**Key learning points/Conclusion:**

We describe a cohort of young people recently transferred from paediatric rheumatology care with the majority having inflammatory arthritis, being female and being on medication. A third had other specialties involved thus requiring greater coordination at the time of transfer. Transitional care preparation appeared effective with the majority seeing professionals independently prior to transfer, self-managing their medication and in receipt of clinic letters copied to them rather than their parents. Documentation of transitional care is important for multidisciplinary care but was sub- optimal with less than half of the records having transition check lists and a minority with formal transfer documentation. With limited clinic time and a childhood onset disease, a brief summary of relevant information including previous medications tried is important for the adult team and core to current transitional care guidance.

The time between last paediatric and first adult appointment has been reported to be important in transitional care and it was reassuring to see this was a median of 4 months. There were higher rates of non-attendance in the young adult clinic compared to paediatrics which could reflect this wide range for continuity and will be an important area for future attention to ensure engagement of young people and the avoidance of lapses in care and potential disease flares. Disease activity was poorly documented in the peri-transfer period however, so it is difficult to comment on prevalence of uncontrolled disease at this time.

Documentation of routine psychosocial screening was better post transfer but may reflect that these appointments are essentially a new patient appointment and therefore reflects the adult team getting to know the young person.

This service evaluation has therefore identified areas of good transitional care practice as well as areas for improvement in this service and will be a useful baseline for future development.

